# Croizat’s form-making, RNA networks, and biogeography

**DOI:** 10.1007/s40656-023-00597-0

**Published:** 2023-11-27

**Authors:** Karin Mahlfeld, Lynne R. Parenti

**Affiliations:** 1 Openlabnz, 5 Imlay Crescent, Wellington Ngaio, 6035 New Zealand; 2 Division of Fishes, National Museum of Natural History, Smithsonian Institution, Washington, DC 20560 USA

**Keywords:** Epigenetics, Differential form-making, Junk DNA–evolution–complex systems, Tectonic calibration

## Abstract

Advances in technology have increased our knowledge of the processes that effect genomic changes and of the roles of RNA networks in biocommunication, functionality, and evolution of genomes. Natural genetic engineering and genomic inscription occur at all levels of life: cell cycles, development, and evolution. This has implications for phylogenetic studies and for biogeography, particularly given the general acceptance of using molecular clocks as arbiters between vicariance and dispersal explanations in biogeography. Léon Croizat’s development of panbiogeography and his explanation for the distribution patterns of organisms are based on concepts of dispersal, differential form-making, and ancestor that differ from concepts of descent used broadly in phylogenetic and biogeographic studies. Croizat’s differential form-making is consistent with the extensive roles ascribed to RNAs in development and evolution and recent discoveries of genome studies. Evolutionary-developmental biology (evo-devo), including epigenetics, and the role of RNAs should be incorporated into biogeography.

For me, Croizat’s contribution is one of liberation. Once we have escaped from the necessity of seeking restricted centres of origin, and from the necessity of plotting routes of dispersal from these centres, and once we have seen the possibilities unfolded by concepts of vicariance and differential form-making then a new world of ideas opens up for us. (Ball, 1975, p. 422)

By imagining that there is a one-to-one relationship between genes and phenotype…biologists lose sight of the role of the environment and polygenic influences in development and evolution. (West-Eberhard, 2011, p. 11)

Although molecular biology, genetics and related special disciplines represent a large amount of empirical data, a practical method for the evaluation and overview of current knowledge is far from realized. (Witzany, 2016, p. 1)

While the story is still unfolding, we conclude that the genomes of humans and other complex organisms are not full of junk but rather are highly compact information suites that are largely devoted to the specification of regulatory RNAs. These RNAs drive the trajectories of differentiation and development, underpin brain function and convey transgenerational memory of experience, much of it contrary to the long-held conceptions of genetic programming and the dogmas of evolutionary theory. (Mattick & Amaral, 2022, p. xii)

## Introduction

In a recent article on the botanist and biogeographer Léon Croizat (1894–1982), and the debates surrounding Croizat’s major works on plant and animal biogeography, *Panbiogeography* (1958), *Principia Botanica* (Croizat, 1960), and *Space, Time, Form: The Biological Synthesis* (Croizat, 1964a), Morrone (2021) expressed concern about the lack of biogeography in Extended Evolutionary Synthesis (EES) debates. Morrone (2021, p. 36) concluded that it is “…perplexing that biogeographic concepts seem to be absent from the discussions on the theoretical framework of the Extended Synthesis … that seems to be more focused on genomics and evo-devo”. It is perplexing since Croizat wrote extensively six decades ago about topics that are central to EES. Yet, the absence of EES from biogeography is a parallel concern, especially with the revival of epigenetics underpinned by the discovery of diverse regulatory RNA networks and virolution (e.g., Villarreal, 2005; West-Eberhard, 2011; Shapiro, 2011, 2021; Ryan, 2019; Mattick & Amaral, 2022). These findings have spurred a new understanding of the function and evolution of genomes but have hardly entered debates in biogeography. Croizat (1964a, p. 6) wrote often about the mechanisms of differential form-making: “The biogeographer is interested in *form-making*, that is, in the process responsible over space in time for the appearance of a certain taxonomic group at a certain point of the map….”. These mechanisms of differentiation include recombination of characters in evolution, not covered by Morrone (2021) but relevant to current debates in modern evolutionary biology and to the seemingly endless disputes concerning the formation of species distributions.

Léon Croizat was prolific and wrote on concepts such as character recombination, differential form-making, environment and inheritance, Lamarckism, Darwinism, dispersal, mutation, selection, macro- versus microevolution, phases of mobilism and immobilism, and so on. Studying Croizat’s works confirms that he read widely and kept abreast of the science and philosophy of the day. Yet modern evolutionary biology ignores Croizat’s work and his emphasis on the discovery and significance of overlapping, repeated distributions, which is the focus of panbiogeography.

Recently, Shapiro and Noble (2021) listed scientists and their discoveries that were either un- or underacknowledged in evolution and who have now come to the forefront of arguments that extend the evolutionary synthesis. They included Richard Goldschmidt (1878–1958) and macroevolution; Boris Mikhailovich Kozo-Polyansky (1890–1957) and evolution by symbiosis or symbiogenesis; Barbara McClintock (1902–1991) and chromosome restructuring after breakage and mobile genetic ‘‘controlling elements’’ (transposons); Conrad Waddington (1905–1975) and epigenetic control of genome function; Roy J. Britten (1919–2012) and repetitive DNA in the genomes of complex organisms; Carl Woese (1928–2012) and Archaea; Lynn Margulis (1938–2011) and symbiogenetic origin of eukaryotic cells; and Stephen Jay Gould (1941–2002) on punctuated equilibrium in the fossil record.

Modern evolutionary theory stresses the contributing roles of multiple inheritance mechanisms to evolution (e.g., Jablonka & Lamb, 2020), which Croizat implicitly accepted in his work more than half a century ago although specific mechanisms were not then well understood. Croizat was familiar with Goldschmidt’s and others cytogenetic work (Croizat, 1964a, pp. 452, 513). He wrote (Croizat, 1964a, p. 709): “The limits between “chromosome” (and its bywords) and “enzymes” wear out exceedingly thin. Vast alterations of forms may be triggered into existence (see, e.g., cleistogamy in plants) by ‘hormonal’ intervention.” His concepts of a non-uniform ancestor (polymorphism) and normal dispersal or range expansion (mobilism) and his view on the roles of geology and vicariance align well with modern evolutionary thought.

In Croizat’s view, evolution is a function of biological structure or form and its spatial and temporal structure, a co-constructed system. In proposing this interrelationship, Croizat presented an evolutionary model that incorporated epigenetics, differential form-making involving structural and adaptive form-making, and a variable pace of speciation that foreshadowed some of the current arguments in molecular evolution. For example, Croizat was adamant that chromosome alterations were not the *cause* but the *result* of evolution (Croizat, 1964a, p. 709), which is consistent with genome inscription as described, for example, by Shapiro (2011, 2021). For Croizat (1964a, p. 12, italics in original), “…*biogeography cannot be extricated from evolution, and the other way around*…”.

### Léon Croizat’s concept of differential form-making

Natural selection was commonly assumed to effect cumulative small mutations. But modern biology shows that different kinds of hereditary inputs determine the phenotype, not just random mutations (Jablonka & Lamb, 2020, p.58). Organisms are remodelled by genetic and epigenetic processes (including non-ancestral horizontal gene transfer from viruses). Sperm, for instance, carry vesicles containing RNAs originating from different organs of the organism. Zygotes, therefore, carry parental genetic and somatic information (Spadafora, 2017). Bursts of genome duplication occurred during early chordate evolution (McLysaght et al., 2002). And numerous proteins that are co-opted for mammalian physiology and development stem from Transposable Elements (inactive relics of former virus invasions/infections) as summarised, for example, by Wells and Feschotte (2020). Genome sequencing has shown that organismal complexity correlates with the number of non-protein coding sequences (C and G value enigmas, Mattick & Amaral, 2022, ch. 7). Likewise, Croizat did not agree with the widespread view that organisms evolved by selection and adaptation alone: “If some of the characters … suggest the influence of the environment, and may be construed accordingly as “ecological” or “selective”, others … do not fall in this same category. It is a common error to interpret character geography … as but “adaptive” and answerable to “environment”, when in reality its roots may be purely structural and so directional” (Croizat, 1964a, p. 111).

Ancestral non-coding conserved regions that do not code for proteins yet show high conservation among vertebrates align with Croizat’s emphasis on the importance of structural form-making, i.e., the existence of widespread types of organisation underpinned by non-protein coding conserved regions, to establish the broad ancestral cosmopolitan patterns from which modern species distributions subsequently evolved. Such molecular structures were described by Aloni and Lancet (2005, p. 115): “Any human sequence that can reliably be aligned to chicken or fish sequence, therefore, strongly suggests functional constraints”. The “freezing” of such DNA sequences suggests that they are involved in regulatory functions that are fundamental to ontogeny and physiology (e.g., Bejerano et al., 2004; Simons et al., 2006; Fedorova et al., 2022).

The abundance and distributions of these genome regions may go a long way towards explaining current distribution patterns of organisms and support Croizat’s ideas of widespread ancestors, evolution on broad fronts, and vicariism, which he deduced from the thousands of plant and animal distributions he had mapped and his morphological analyses (e.g., Croizat, 1964b). Structural constraints on the drift of different sequences are apparently much greater than previously thought given the current knowledge of genomes. Croizat’s structural form-making as a basic biogeographic principle, fundamental to understanding distributional patterns of organisms, is consistent with the widespread occurrence of RNA networks(e.g., Croizat, 1958, p. 822; Croizat, 1964a, pp.627–672 for various comments on natural selection, teleology vs. biology, orthogenesis, structure and function, and vicariism). Croizat observed the “orderly interrelationships of space and form” (Croizat, 1964a, p. 630) and that “geologic and geographic change do promote form-making” (Croizat, 1964a, p. 643) and set “structural and evolutive premises” (Croizat, 1964a, p. 647). These premises are now well documented in the orchestrated interactions of RNA networks enabling ontogeny and evolution, hybrid processes that involve genetic and epigenetic elements.

As an example, Croizat used the evolution of scorpions which evolved from a certain structural level exhibited by all ancestral pre-scorpions and vicariant form-making through adaptations to local conditions and geological changes. In his view, form-making was “a diffusive, simultaneous process of deployment” (Croizat, 1964a, p. 200) with no particular origin in the sense of appearing out of nowhere but “structurally pre-conditioned during a stage of evolution” concerning pre-scorpion populations worldwide more or less simultaneously (p. 218) illustrating the concept of ancestral cosmopolitanism. It explains why an organism lives within a particular geographic setting and not any other. Structural evolution, such as ancestral non-coding conserved regions that put structural constraints on the drift of different types of sequences without telos (i.e., not for a preconceived benefit, purpose, or profit), is the primary driver of distributions.

Croizat (1964a) illustrated the interplay of structural and adaptive form-making (differential form-making) further with the example of the New Zealand air-breathing gastropod *Cytora pallida* (Hutton, 1883) by quoting New Zealand zoologist John Morton in a footnote on p. 218: “[Species of *Cytora*] Have accomplished the transition from sea to a land habitat *with relatively few modifications of their primitive structure* …. the air-filled pallial cavity remains widely open anteriorly and there is, properly speaking, no development of a lung, respiration taking place merely through the smooth vascularised epithelium of the pallial roof; *gill filaments are lost and there are no folded respiratory lamellae*” (Croizat’s italics). And Croizat followed up in the same footnote: “Noteworthy is the implicitly *stress on structure* in the text quoted; a tendency acting in the direction of structural reduction of gill filaments and folded respiratory lamellae would of course go far in explaining why this mollusc became “adapted” to “air” (p. 218).

Early examples of structural form-making and vicariism were provided by Ludwig K. Schmarda and Paul and Fritz Sarasin. Schmarda (1853, p. 91–93) described cases of similar looking species in similar habitats in different continents. In Croizat’s view, this is due to the “…very same background of evolutionary process” … “the release of like ‘mutations’ in the wake of an ‘oriented’ tendency active in places geographically so remote” (Croizat, 1964a, p. 654–655). Sarasin and Sarasin (1899, p. 228) doubted that “chains” of closely related species (*Formenketten*) of terrestrial molluscs on Sulawesi could be explained sufficiently by natural selection alone. Instead, the distribution of widely shared, developmental and evolutionary processes of the molluscs (broad front structural form-making producing a widespread ancestor) and local adaptive form-making in response to geologic and geographic change can explain *Formenketten*. Another recent example that is compatible with Croizat’s concept of structural form-making is provided by Moelling et al. (2017) who describe the same structures and mechanisms involving RNase H and its role in building immune systems in organisms.

## Vicariance and dispersal in a changed evolutionary landscape

### A better understanding of genome landscapes

Reconstructing evolutionary history based on differences among particular molecular markers and average rates of substitutions arguably captures only a fraction of evolutionary change and may not always be reliable, even less so if these characters are not evaluated in the overall genome landscape in which they reside. Differences alone do not explain the history or nature of such differences. The assumption that amino acid or nucleotide substitutions accumulate at reasonably constant rates across taxa over evolutionary timescales unperturbed by other processes in the genome such as repair mechanisms, for example, is being abandoned (Yi, 2013). To improve the application of molecular clocks, it is necessary to understand the processes that lead to mutations and allele substitutions and how these are accelerated, avoided, or repaired (Yi, 2013). Shapiro (1999, p. 172) wrote: “A 21st century view of evolution will incorporate a more informational perspective on the structure and operation of genetic systems.” […] “Darwinian gradualism [i.e., random mutations and selection] cannot explain the origin of complex integrated systems needed for adaptation or survival” (Shapiro, 1999, p. 176), and “organisms have a far more powerful evolutionary potential to generate integrated genomic networks and ensure the survival of their descendants than predicted by current theories of gradualism and random mutation” (Shapiro, 1999, p. 177–178). This ability of organisms to reconfigure their genomes and thereby the effect of selection pressures makes it difficult to determine reliable molecular clock rates.

Genome research has unearthed RNA regulatory networks at all levels of life and has overturned many of the textbook concepts on which evolutionary and phylogenetic studies are based, e.g., novelty through selection of random mutations, the Weismann Barrier, and the directional flow of genetic information (e.g., DNA→RNA→protein) or neutral selection of the large proportion of the non-protein coding sequences of genomes. Introns, which have been used as a proxy to enforce the concept of neutral selection (introns are not incorporated into the mature mRNAs and were once thought as more likely to be neutral regarding selection), are functional areas of the genome (Mattick & Amaral, 2022). The amount of exaptation (where a feature is co-opted for a function for which it was not originally adapted or selected) of non-protein coding sequences, once thought of as evolutionary “junk” but now known to be relevant in all processes of replication, transcription, translation, immunity, repair, and novelty, is nowadays much better appreciated. A lot of this “junk” was epigenetically triggered, imprinted, and inscribed. The assumption that mutations are random and neutral, once premises in the concept of molecular clocks, has been questioned for some time (e.g., Noble, 2006).

### Different perspectives on dispersal

Molecular clock dating based on fossil calibration is a popular, yet controversial, method to decide if a particular distribution results from chance dispersal or vicariance. Better understanding of how genomes operate and the hybrid nature of development and evolution involving genetics, epigenetics, and virolution can improve the application of molecular clocks in biogeography. By far the most common method to attach an absolute time scale to a molecular tree is calibration based on the assumed maximum age of fossils, regardless of the criticisms this method has received (e.g., Blair & Hedges, 2005; Nelson & Ladiges, 2009; Wilke et al., 2009; Heads, 2012a; Wang & Mao, 2015; Wilf & Escapa, 2015; Klopftstein, 2021). A survey by Hipsley and Müller (2014; quoted by Heads, 2017, p. 59) of 613 papers, published between 2007 and 2013, showed that just 15% of them used tectonic calibration.

The use of fossil age calibrated molecular clocks as arbiters between distributions due to chance dispersal or in situ structural and adaptive evolution (vicariance) has been largely counterproductive in prematurely aborting vicariance-based explanations rather than encouraging investigations into why molecular clock dated phylogenies conflict with vicariance. The competing concepts of vicariance and chance dispersal credit geology with entirely different roles: vicariance is integrated with geology, Croizat’s “Earth and Life evolve together”, chance dispersal assumes Earth is a stage over which life moves, independent of geology, and at random.

It is important to distinguish long-distance chance dispersal from dispersal within a species’ natural vagility, even if that covers long distances, because it may well be within the metapopulation survival range of species (past and present) and not require accidental transport (see also Cain, 1944). Many studies endorse chance dispersal or jump dispersal as an explanation for the development of species ranges because molecular clock dated phylogenies rule out a particular vicariance explanation. In contrast, Croizat (1958, 1964a) often concluded his biogeographic accounts with the phrase “*Dispersal forever repeats*”, emphasising regularities and patterns he had observed through mapping distributions across taxa. Dispersal as interpreted by Croizat was the record of form-making over broad fronts in a series of geological and climatic events that led to the fragmentation and diversification of a former widespread ancestor like repeated blows shattering a piece of glass. By mapping the distributions of taxa of a group or clade, this differentiation of widespread ancestors across a landscape would be recorded on the map. The taxa are not interpreted to have migrated across a broad landscape. Instead, they were “…*formed there out of ancestors that were already there, step after step, blow by blow*” (Croizat, 1964a, p. 209, italics in original). For Croizat (1964a, p. 13) “…*geographic distribution* holds the record, *dispersal* interprets it.” This is contrary to chance dispersal that forms no pattern. Ebach and Williams (2016) called chance dispersalism “neodispersalism” to contrast it with Croizat’s meaning.

Proponents of the molecular clock analyses in biogeography rarely include distribution maps and may not be swayed by distributional evidence, as noted by McCarthy (2005, p. 6): “Despite the efficacy of distributional analyses … a number of researchers have abandoned this glorious tradition of biogeography and now use everything *except* [italics in original] distributional facts when fashioning distributional explanations. The result is a recent spate of hypotheses of cross-ocean rafting events of terrestrial vertebrates and pattern of convenient fossil absences—all of which are required to maintain fashionable geological and molecular-clock assumptions.” The hypothesis, dispersal from a centre of origin as inferred from a phylogeny, is treated as the evidence for the hypothesis which means it is never tested, only replaced with a new hypothesis (see also Parenti & Ebach, 2013; Parenti, 2017). And although phylogenetic breaks are often spatially correlated with geological or climatic events, geological breaks are frequently dismissed as too old to be relevant because molecular dating techniques reject older ages of lineages (Heads, 2012a).

In this context, we note a recent analysis of the distribution of fossil taxa and their palaeoclimate that supports the conclusion that vertebrates did not cross the relatively narrow Palaeozoic marine Ural Seaway (between Siberia and Baltica) via random or ‘sweepstakes’ dispersal. Brikiatis (2020) investigated the impact of arido-eustatic cycles on the Palaeozoic evolution of vertebrates and copepods and constructed a vicariance model that was highly predictive of fossil distributions and in agreement with sea level stands and paleoclimatic records. He concluded that vicariance, i.e., in situ evolution, was the predominant mode of vertebrate evolution (also supported by recent, more accurate radioisotopic dating of fossiliferous rock containing early synapsid taxa).

Likewise, Nelson (2006); Heads (2012b) and others gave a different perspective on the Hawaiian Islands and their biota, which are often used as an unarguable example of island colonisation by long-distance, chance dispersal. Heads (2012b, 2018) drew on evidence from seafloor geology and applied a metapopulation survival model in contrast to long-distance chance dispersal and extinction for the evolution of the Hawaiian biota.

Vicariance has been dismissed as a biogeographic mechanism both historically (e.g., du Toit, 1944) and in recent literature (e.g., Heads, 2012b, 2017), which, in principle, is also a dismissal of differential form-making on broad fronts since vicariance is its natural consequence. Yet, RNA networks with their widely shared structures, mechanisms and processes support broad front, differential form-making.

## Concluding remarks

Many modern biologists are unfamiliar with Croizat’s work. Developmental biology, physiology, microbiology, or medicine rarely concern themselves with biogeography. Investigations are largely at a molecular to intermediate cellular or organismal level and not in a biogeographic context. Croizat was interested equally in biological form and geographic distributions, which led him to develop panbiogeography. Raising awareness of his work, we hope, will close the gaps between evo-devo, EES, and biogeography. Croizat embraced different evolutionary processes affecting speciation (his continual reference to differential form-making, polytypical and polytopical processes) and can be regarded as one of the original champions of the modern evolutionary biology movement (see Vane-Wright, 2022).

The conflicting views between dispersal and vicariance biogeographers might be easier to resolve given new genome discoveries and with the renaissance of epigenetics rather than accepting chance dispersal because popular interpretations of molecular clocks demand so. Genome inscription is an active physiological process at all scales (Shapiro, 2011, 2021). Distinguishing between genetic and epigenetic inheritance once reaffirmed a one-way flow of genetic information; at the same time, epigenetic inheritance was regarded as a transient phenomenon, only lasting for a few generations. Today, the dynamics between genetic and epigenetic inheritance are better understood in terms of RNA-mediated epigenetic inheritance and RNA-templated DNA repair (Mattick & Amaral, 2022). The implications of modern genome research for current approaches in systematics and biogeography have not yet been widely considered. We anticipate that they will shed a different light on the arguments against the contributions of Croizat and New Zealand panbiogeographers, which have been narrated by Morrone (2021). We look forward to the recognition of the relevance of this research and its incorporation into modern discussions of evolutionary theory and biogeography.
